# The selective inhibitor of nuclear export (SINE) verdinexor exhibits biologic activity against canine osteosarcoma cell lines

**DOI:** 10.1111/vco.12680

**Published:** 2021-01-26

**Authors:** Justin T. Breitbach, Darian S. Louke, Savannah J. Tobin, Mauria R. Watts, Alexander E. Davies, Joelle M. Fenger

**Affiliations:** ^1^ Department of Veterinary Biosciences College of Veterinary Medicine, The Ohio State University Columbus Ohio USA; ^2^ Department of Veterinary Clinical Sciences College of Veterinary Medicine, The Ohio State University Columbus Ohio USA

**Keywords:** comparative oncology, dog, doxorubicin, XPO1

## Abstract

Verdinexor (KPT‐335) is a novel orally bioavailable selective inhibitor of nuclear export (SINE) compound that inhibits the function of the nuclear export protein Exportin 1 (XPO1/CRM1). In the present study, we sought to characterize the expression of XPO1 in primary canine osteosarcoma (OS) tumour samples, OS cell lines and normal osteoblasts and evaluate the in vitro activity of verdinexor alone or in combination with doxorubicin. Canine OS cell lines and a subset of primary OS tumours showed increased XPO1 transcript and protein expression as compared with normal canine osteoblast cells. All canine OS cell lines exhibited dose‐dependent growth inhibition and increased caspase 3,7 activity in response to low nanomolar concentrations of verdinexor (IC_50_ concentrations ranging from 21 to 74 nM). Notably, growth inhibition of normal canine osteoblast cell lines treated with verdinexor was observed at high micromolar concentrations (IC_50_ = 21 μM). The combination of verdinexor and doxorubicin resulted in potent inhibition of cell viability and demonstrated synergetic activity in three canine OS cell lines. Concordantly, OS cell lines showed increased γH2A.X foci following treatment with doxorubicin and recovery in verdinexor compared with cells treated with doxorubicin and recovered in normal media for 24 hours. These findings demonstrate that verdinexor has biologic activity against canine OS cell lines at physiologically relevant doses and suggest that XPO1 inhibition in combination with standard doxorubicin treatment offers promising potential for chemotherapeutic intervention in canine OS.

## 1 INTRODUCTION

With an estimated incidence of >10 000 new cases per year, osteosarcoma (OS) is the most common primary bone tumour in dogs.^1^ Despite aggressive treatment involving limb amputation and systemic chemotherapy, 90% of dogs succumb to chemotherapeutic resistant metastatic disease.^2^ Similar to canine OS, 30% to 40% of humans with OS die from metastatic disease despite treatment.^3^, ^4^, ^5^ Targeted therapeutics and immunotherapy have revolutionized survival outcomes in a variety of cancers; however, to date, these have not translated in improved outcomes in either human or canine OS patients.^6^, ^7^, ^8^, ^9^, ^10^, ^11^, ^12^, ^13^ As systemic chemotherapy remains the backbone for treatment of OS metastases, the development of combinational treatments with novel and more effective agents is necessary for this disease.

The exportin‐1 protein (XPO1 or CRM1) functions in mammals to regulate the transport of RNA and protein via the nuclear‐cytoplasmic transport system.^14^, ^15^ XPO1 is the major nuclear export receptor from the Karyopherin β protein family that interacts with the leucine‐rich nuclear export signal (NES) of cargo proteins for nuclear‐cytoplasmic export. Transport of the XPO1‐cargo protein complex is mediated by RanGTP which is bound with the XPO1‐cargo protein complex and passively diffuses into the cytoplasm through the nuclear pore complex down a RanGTP gradient.^16^ Dysregulation of this process has been implicated in tumorigenesis as aberrant trafficking of tumour suppressor and regulatory proteins modifies apoptosis and cell proliferation pathways. Of the ~200 NES containing cargo proteins shuttled by XPO1, the tumour suppressor and regulatory growth proteins p53, p21, RB, survivin and FOXO are several involved in this imbalance.^17^ Increased concentrations of XPO1 have been identified in a number of solid and hematologic malignancies and are associated with poor prognosis.^18^, ^19^, ^20^, ^21^, ^22^ As such, restoration of nuclear‐cytoplasmic protein homeostasis via XPO1 inhibition may represent a viable therapeutic strategy for human and canine cancers.

Selinexor and verdinexor, two selective inhibitors of nuclear export (SINE) compounds that specifically inhibit the binding of cargo proteins to XPO1, have shown clinical benefit in human and canine cancer patients, respectively.^17^, ^23^ Selinexor (tradename XPOVIO) was recently granted approval by the FDA for the treatment of patients with relapsed or refractory multiple myeloma in combination with dexamethasone.^24^ In addition to its efficacy in the treatment of hematologic malignancies, selinexor has shown in‐vitro biological activity in human OS cell lines and robust in vivo antitumor activity was observed in a variety of preclinical sarcoma animal models.^25^, ^26^ In a phase IB clinical trial evaluating single‐agent treatment with selinexor in patients with refractory bone or soft tissue sarcomas, 33% of patients experienced stable disease for 4 or more months.^27^ Increased expression of XPO1 in OS tissues was correlated with a reduced progression‐free survival and overall survival, suggesting that therapeutics targeting XPO1 may be effective in OS treatment.^19^

Prior studies investigating the in vitro activity of verdinexor in canine cancer cells demonstrate that verdinexor inhibits cell proliferation in canine lymphoma, melanoma, transitional cell carcinoma, and mammary carcinoma cell lines.^28^, ^29^, ^30^ A phase I clinical trial in dogs with spontaneous cancers established safe dosing regimens at physiological achievable concentrations of verdinexor.^29^ Subsequent phase II trials evaluating treatment with verdinexor in dogs with non‐Hodgkin lymphoma found that verdinexor exhibited biologic activity with an overall objective response rate of 37%.^23^ While these studies with verdinexor show promise for its use in the treatment of canine lymphoma and have provided important pharmacokinetic data in canines, the potential efficacy of verdinexor in the treatment of canine OS remains unclear.

The purpose of this study was to confirm the relevance of XPO1 as therapeutic target in canine OS and evaluate the activity of verdinexor alone and in combination with the cytotoxic chemotherapeutic drug, doxorubicin in canine OS cells. These data will serve as the foundation for potential future clinical studies investigating verdinexor in dogs with OS.

## 2 MATERIALS AND METHODS

### 2.1 Reagents

Verdinexor was graciously donated by Anivive Lifesciences (Long Beach, California). Stock solutions of verdinexor were prepared in 100% DMSO, stored at −20°C, and diluted in the indicated culture medium for cell line treatments.

### 2.2 Cell lines and primary samples

The canine cell lines OSCA‐8, OSCA‐16, OSCA‐40 were provided by Dr Jaime Modiano (University of Minnesota, Minneapolis, Minnesota). The Abrams cell line was provided by Dr Doug Thamm (Colorado State University, Fort Collins). The D17 cell line (Cat.# ATCC CCL‐183) was purchased from the American Type Culture Collection (Manassas, Virginia). Normal canine osteoblasts (Cat.#Cn406‐05) were obtained from Cell Applications, Inc. (San Diego, California). Cell lines were cultured in Gibco (Thermo Fisher Scientific) RPMI 1640 (OSCA‐8, OSCA‐16), DMEM medium (Abrams, OSCA‐40, D17), or CnOb growth medium (Cell Applications, Cat.#Cn417‐500). Media was supplemented with 10% foetal bovine serum, 1% l‐glutamate, 1% penicillin/streptomycin, 1% 4‐(2‐hydroxyethyl)‐1‐piperazineethanesulfonic acid, 1% non‐essential amino acids, and 1% sodium pyruvate (Gibco). Cells were cultured in a humidified incubator containing 5% CO_2_ at 37°C. Cells were routinely tested for mycoplasma and if positive, treated with Plasmocin (Cat.#ant‐mpt‐1, Invivogen) and re‐tested to ensure complete eradication.

Canine OS tumours were collected from patients treated at The Ohio State University College of Veterinary Medicine Veterinary Medical Center (OSU‐CVM VMC) in accordance with established hospital protocols and approved by the Institutional Animal care and Use Committee (IACUC #2010A0015). Fresh tumours were flash frozen in liquid nitrogen and confirmed to be OS by board certified veterinary anatomic pathologists at OSU‐CVM VMC.

### 2.3 Cell line validation statement

Six cell lines were used in this study, and all cell lines were previously established and validated. Two cell lines were purchased directly from commercial culture collections, as outlined above. Cells were not subjected to species verification testing; however, morphology under light microscopy and growth kinetics remained consistent throughout the experiments.

### 2.4 RNA isolation, cDNA synthesis, and qRT‐PCR

RNA was extracted from canine OS cell lines and tumours using TRIzol reagent (Invitrogen) and purified using the RNeasy Mini Kit (Qiagen, Cat.#74 104) according to manufacturer's instructions. cDNA was made from 2 μg of total RNA using Superscript III (Invitrogen). Published canine XPO1 primers and 18S primers were used with Fast SYBR Green Master Mix (Applied Biosciences) and qRT‐PCR was performed using the Applied Biosystems StepOne Plus Detection System (Applied Biosystems, Foster City, California).^30^ Relative expression of XPO1 was determined using the comparative threshold cycle method and normalized to the endogenous housekeeping gene 18S.^31^ All reactions were performed in triplicate and three independent experiments were performed in OS cell lines.

### 2.5 Immunoblotting

OS cells were collected, washed with 1X Dulbecco's phosphate‐buffered saline (Gibco) and re‐suspended in complete lysis buffer consisting of 20 mM Tris‐HCL pH 8.0, 137 nM NaCl, 10% glycerol, 1% IPEGAL CA‐630, 10 mM ethylenediaminetetraacetic acid, 1 mg/mL aprotinin, 1 mg/mL leupeptin, 1 mg/mL pepstatin A, 1 mM phenylmethylsulphonyl fluoride, 1 mM sodium orthovanadate, and 10 mM sodium fluoride for 1 hour at 4°C for protein extraction. Frozen tumours were pulverized and re‐suspended in complete lysis buffer for 1 hour at 4°C. Extracted protein was quantified using the Bradford assay and 75 μg protein was separated by SDS‐PAGE and transferred to PVDF membranes. Membranes were blocked in TBST containing 5% non‐fat dry milk for 1 hour at room temperature and incubated overnight with anti‐XPO1 (5G3, Invitrogen, Cat.#MA5‐27879, 1:40 000 dilution) at 4°C. Membranes were incubated in horseradish peroxidase linked anti‐mouse secondary antibody (Cell Signalling, Cat.#7076), washed, and exposed to substrate (SuperSignal West Dura Extended Duration Substrate, Pierce, Rockford, Illinois). Blots were stripped (Restore, Pierce), washed, and re‐probed for β‐actin (8H10D10, Cell Signalling, Cat.#3700, 1:20000 dilution) overnight at 4°C. Band intensities were calculated with ImageJ software (NIH, Bethesda, Maryland) and relative intensity of XPO1 was determined by dividing by β‐actin.

### 2.6 Cell proliferation

The effect of verdinexor on canine OS cell viability was assessed with the CyQUANT Cell Proliferation Assay Kit (Molecular Probes, Eugene, Oregon). 1 × 10^3^ cells were seeded in 96‐well plates and incubated in media overnight. Cells were treated with 0.1% DMSO (control) or varying concentrations of verdinexor (0.001‐10 μM) for 96 hours. Media was removed after 96 hours and plates were frozen overnight at −80°C. Fluorescence was measured with the UV Spectromax M2 plate reader (Molecular Devices, Sunnyvale, California) with 485 nm excitation/530 nm emission detection. Cell proliferation was calculated as a percentage of the control wells. All treatments were evaluated in triplicate in a least three independent experiments. The concentration of verdinexor that inhibited cell viability by 50% (IC_50_) was determined using the GraphPad Prism v8.3.0 (GraphPad Software, Inc, La Jolla, California).

Drug combination studies were completed with verdinexor and doxorubicin in the D17, OSCA‐40, and Abrams cell lines using the fixed‐ratio method. Combination index (CI) was calculated with the CompuSyn software (ComboSyn, Inc.) to determine synergistic effects using the median‐effect method of Chou and Talalay.^32^ CI = 1, additivity; CI < 1, synergism; CI > 1, antagonism.

### 2.7 Detection of caspase 3/7 activity

Caspase‐3/7 pathway activation following verdinexor treatment was measured by the SensoLyte Homogeneous AMC Caspase‐3/7 Assay Kit (Anaspec Inc, San Jose, California). 1 × 10^3^ OS cells were seed in 96‐well plates and incubated with media containing either 0.1% DMSO (control) or 1 or 10 μM concentrations of verdinexor for 48 hours. Caspase‐3/7 substrate solution was added to all wells and incubated in 5% CO_2_ at 37°C for 18 hours. Fluorescence was measured on the UV Spectromax M2 plate reader (Molecular Devices) using 354 nm excitation/442 nm emission detection. All treatments were evaluated in triplicate in a least three independent experiments.

### 2.8 Immunofluorescence

4.0 × 10^3^ canine OS cells were plated in 96‐well collagen covered glass bottom plates and incubated overnight. For γH2A.X experiments, cells were incubated in media containing 0.1% DMSO, 1 μM doxorubicin for 2 hours, 1 μM verdinexor for 48 hours, or 1 μM doxorubicin for 2 hours followed by recovery in 1 μM verdinexor or 0.1% DMSO for 48 hours. Media was aspirated, cells were fixed with 4% paraformaldehyde and permeabilized with 1% Triton‐X. Cells were blocked in blocking buffer (1× PBS/2% BSA/0.1% Triton‐X) for 60 minutes and incubated with anti‐γH2A.X (Mouse mAb anti‐phospho‐Histone H2A.X [Ser 139] clone JBW30, Sigma‐Aldrich) at a dilution of 1:500 overnight at 4°C. Secondary donkey anti‐mouse antibody (Cat.#A‐21202, Alexa Fluor 488, Invitrogen) was applied for 1 hour. Cells were stained with DAPI for nuclear visualization and γH2A.X fluorescence was detected by immunofluorescence microscopy using a Nikon Ti2E microscope fitted with a Prime 95B camera. Data analysis was performed using custom MATLAB software (MathWorks, Natick, Massachusetts).^33^

### 2.9 Statistics

Data analysis was performed using the software program GraphPad Prism v8.3.0 (GraphPad Software, Inc). Normality of the data was assessed by the Shapiro‐Wilk and/or the D'Agostino & Pearson tests. All experiments were performed three times in at least three independent experiments unless noted elsewhere. A one‐way ANOVA with Dunnett's multiple comparison test was used to evaluate differences in apoptosis and XPO1 gene expression. Values of *P* < .05 were considered statistically significant.

## 3 RESULTS

### 3.1 XPO1 is overexpressed in a subset of primary canine osteosarcoma tumours and canine osteosarcoma cell lines

**FIGURE 1:**
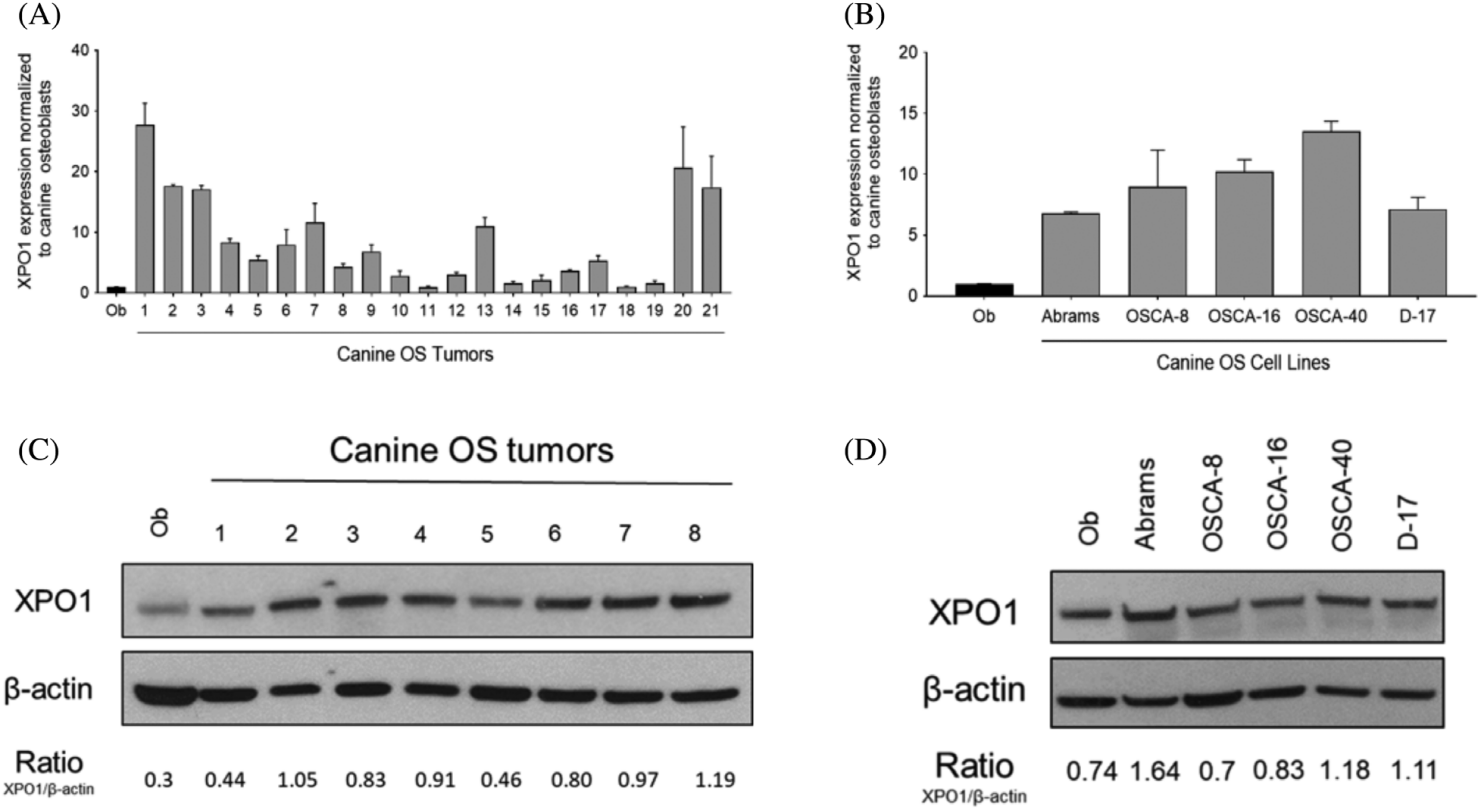
XPO1 expression in canine osteosarcoma (OS) tumours and cell lines. (A) Quantitative real‐time PCR of canine OS tumour samples and cell lines. (B). Western blot demonstrating XPO1 expression in canine OS tumour samples and cell lines. Densitometry band quantification for XPO1 and the corresponding β‐actin were performed using the ImageJ software. Error bars = SD

XPO1 transcript expression was quantified via qRT‐PCR in 21 primary canine OS tumours and XPO1 protein expression was assessed by Western blotting in eight primary canine OS tumours to determine if XPO1 represents a relative target for therapeutic intervention. XPO1 is overexpressed in the majority primary OS tumours at the RNA and protein level when compared with normal canine osteoblasts (Figure 1A). XPO1 expression was subsequently evaluated in five well‐established canine OS cell lines and we found that XPO1 is overexpressed in the majority of cell lines when compared with normal canine osteoblasts (Figure 1B). These data indicate that XPO1 is overexpressed in a subset of primary canine OS tumours and canine OS cell lines and suggest that therapeutic strategies targeting XPO1 may have efficacy in the treatment of canine OS.

### 3.2 Verdinexor inhibits cell proliferation in canine osteosarcoma cell lines

**FIGURE 2:**
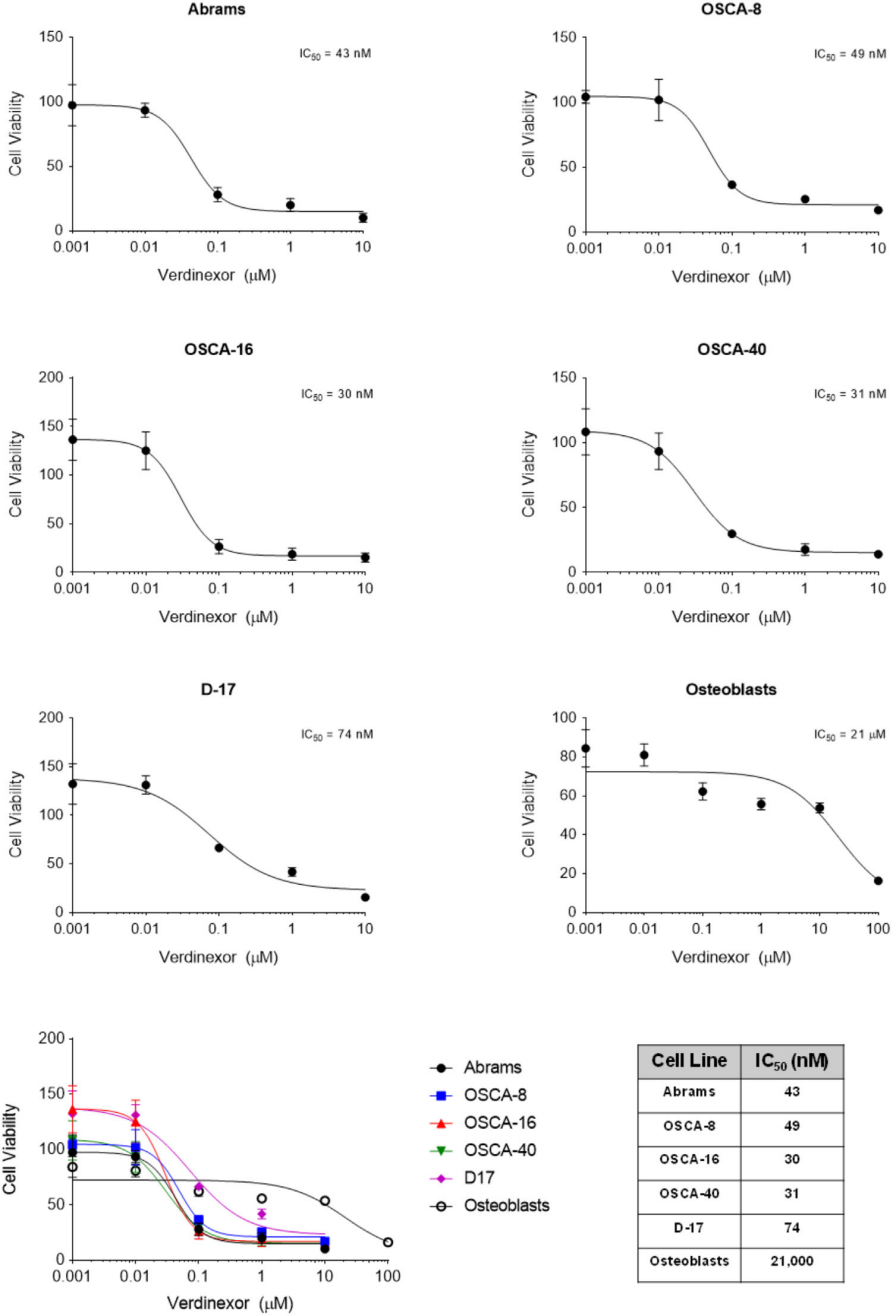
Anti‐proliferative effects of verdinexor in canine osteosarcoma (OS) cell lines and normal osteoblasts. Canine OS cell lines and normal osteoblasts were treated with DMSO or increasing concentrations of verdinexor for 96 hours. Values listed are a percentage of DMSO control. Experiments were performed in quadruplicate and repeated three times. Error bars = SD [Colour figure can be viewed at wileyonlinelibrary.com]

Canine OS cell lines and normal canine osteoblasts were treated with increasing doses of verdinexor to determine whether verdinexor was capable of inhibiting cell proliferation. Cells were treated with 0.001, 0.01, 0.1, 1, 10 μM verdinexor for 96 hours and proliferation was determined using the CyQUANT assay. Figure 2 depicts dose dependent decreases in cell proliferation in all canine OS cell lines. The IC_50_ values for the canine OS cell lines were in low nanomolar concentrations, ranging from 30 to 74 nM. Interestingly, the IC_50_ concentration of verdinexor for normal canine osteoblasts was 21 μM, indicating that normal osteoblasts are significantly less sensitive to verdinexor compared with canine OS cell lines (Figure 2).

### 3.3 Verdinexor enhances caspase 3/7 mediated apoptosis in canine osteosarcoma cell lines

**FIGURE 3:**
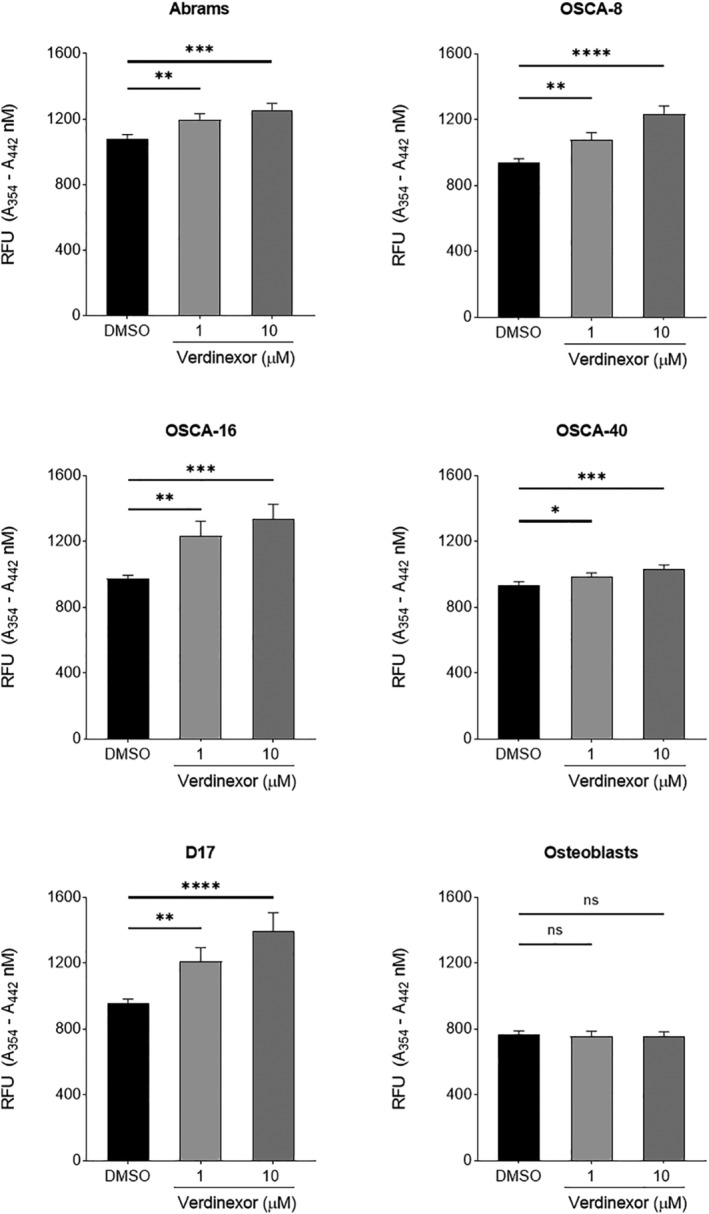
Verdinexor induces apoptosis in canine osteosarcoma (OS) cell lines. Canine OS cell lines and normal canine osteoblasts were treated with 0.1% DMSO, 1 μM, or 10 μM for 48 hours and caspase 3/7 activity was measured using the SensoLyte Homogeneous AMC Caspase ‐ 3/7 Assay Kit. Presented data are an average of four replicates from four independent experiments ±SD. Data were analysed using a one‐way ANOVA with Dunnett's multiple comparisons test. *P < .05, **P < .01, ***P < .001, ****P < .0001

To determine the ability of verdinexor to induce apoptosis in canine OS cell lines, OS cell lines were treated with either 0.1% DMSO, 1 μM verdinexor, or 10 μM verdinexor for 48 hours and caspase‐3/7 activity was measured. Verdinexor induced statistically significant dose‐dependent increases in caspase‐3/7 enzymatic activity in all canine OS cell lines treated, consistent with activation of the intrinsic apoptotic pathway (Figure 3). Normal canine osteoblasts treated similarly with verdinexor did not induce statistically significant increases in caspase‐3/7 enzymatic activity when compared with DMSO treated cells. These data demonstrate that verdinexor enhances apoptosis in canine OS cells while having minimal effect on caspase‐3/7 activity in normal canine osteoblasts at all concentrations evaluated (Figure 3).Concordantly, canine OS cells displayed enhanced sensitivity to verdinexor when compared with normal osteoblasts (Figures 2 and 3).

### 3.4 Verdinexor reduces XPO1 protein expression resulting in a compensatory increase in XPO1 mRNA expression in canine osteosarcoma cell lines

**FIGURE 4:**
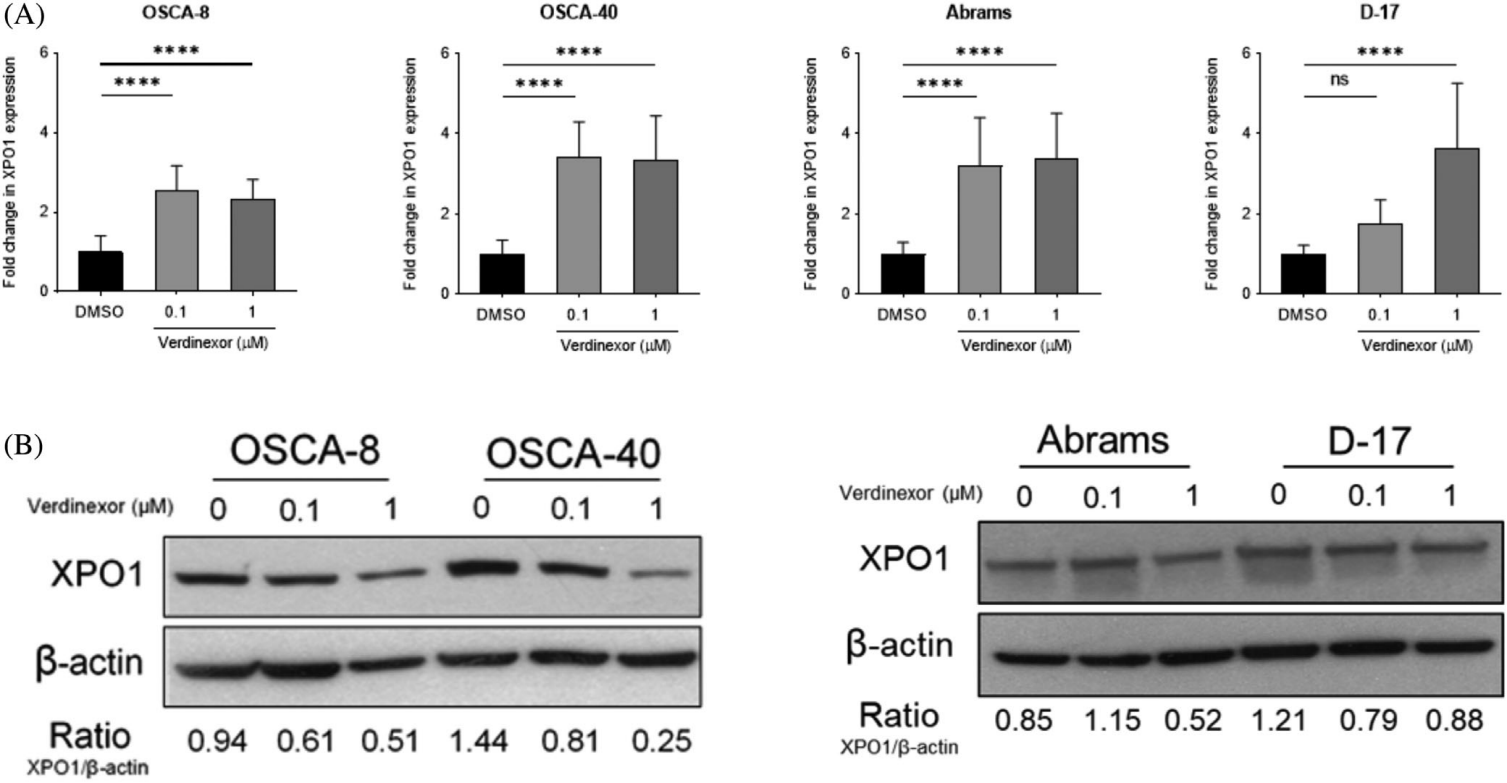
Effects of verdinexor on XPO1 mRNA and protein expression in canine osteosarcoma (OS) cell lines. Canine OS cell lines were treated with 0.1% DMSO, 0.1 μM, or 1 μM for 24 hours and RNA and protein was collected. (A) XPO1 expression measured via quantitative real‐time PCR in canine OS cells treated with verdinexor for 24 hours. Data were analysed using a one‐way ANOVA with Dunnett's multiple comparisons test. *P < .05, **P < .01, ***P < .001, ****P < .0001. (B) Western blotting for XPO1 was performed on canine OS cells treated with verdinexor for 24 hours. Error bars = SD

Previous studies using human cancer cell lines and canine melanoma cell lines have shown that inhibition of XPO1 with SINE compounds can result in a compensatory upregulation of XPO1 mRNA as a result of XPO1 protein loss.^28^, ^34^ To assess if canine OS cells responded similarly, we treated four cell lines with 0.1% DMSO, 0.1 μM verdinexor, or 1 μM verdinexor for 24 hours and evaluated XPO1 expression via quantitative real‐time PCR and Western blotting (Figure 4). Similar to previously published results, we found that treatment with verdinexor caused a reduction in XPO1 protein expression.^35^, ^36^ Additionally, XPO1 mRNA was significantly increased in all cell lines tested. These data support the notion that inhibition of XPO1 with verdinexor in canine OS cells results in reduced XPO1 protein levels with a compensatory increase in XPO1 mRNA.

### 3.5 Combinatorial treatment with verdinexor and doxorubicin results in synergistic inhibition of cell proliferation in canine osteosarcoma cell lines

**FIGURE 5:**
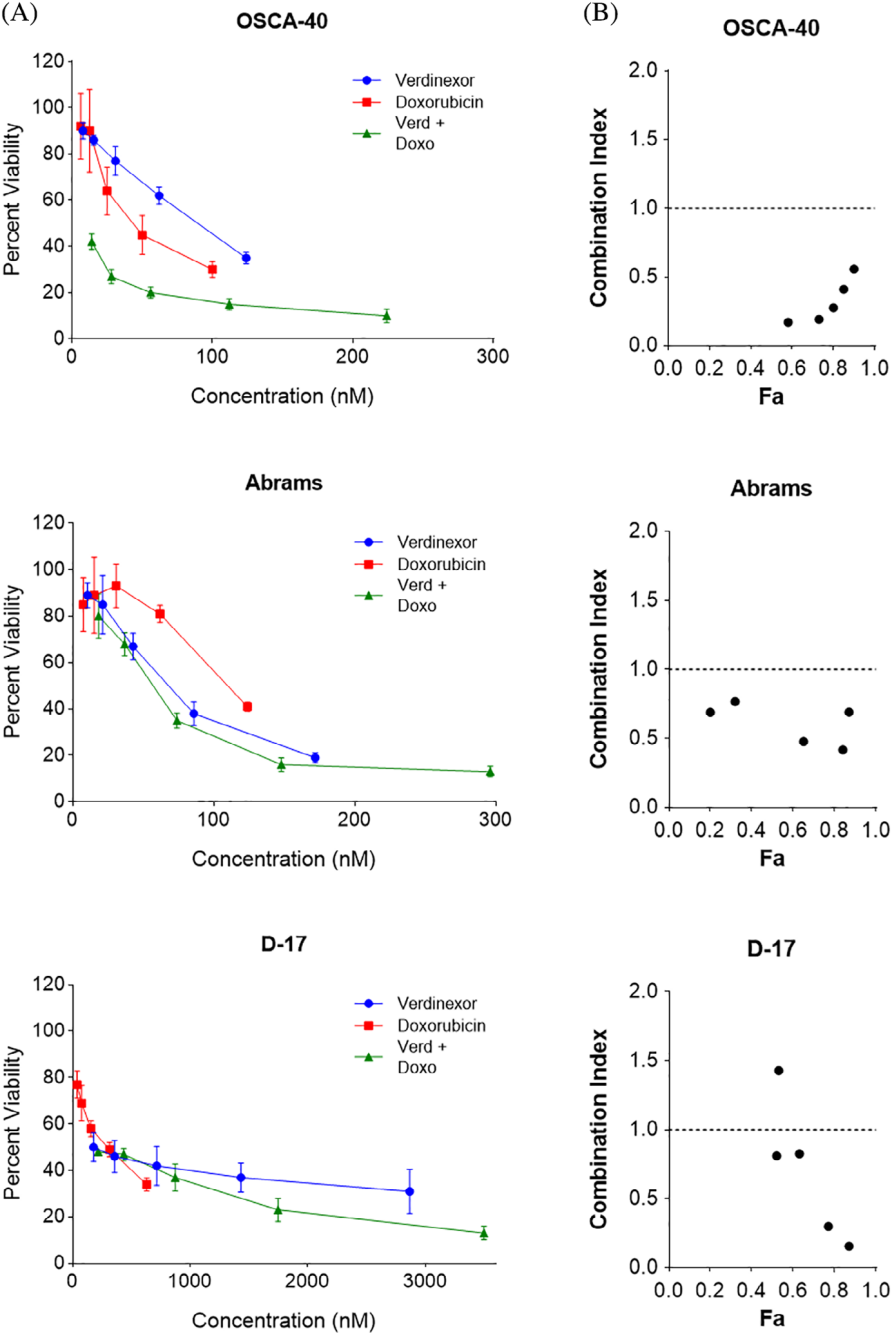
Verdinexor exhibits synergy with doxorubicin in canine osteosarcoma (OS) cell lines. (A) Proliferation in canine OS cell lines exposed to increasing concentrations of verdinexor, doxorubicin, or a combination of doxorubicin and verdinexor are displayed as a fraction of an untreated control after exposure to each agent for 96 hours ±SD. Experiments were performed in triplicate and repeated three times. (B) Combination indices (CI) derived from the aforementioned experiments. A CI equal to 1 is consistent with an additive effect, a CI < 1 is consistent with synergism, and a CI > 1 is consistent with antagonism [Colour figure can be viewed at wileyonlinelibrary.com]

As cytotoxic chemotherapy remains the standard of care for treatment of microscopic metastatic disease, combinational studies with verdinexor and the chemotherapeutic drug doxorubicin were performed in three canine OS cells lines to determine whether combinational treatment further reduced cell proliferation. Abrams, OSCA‐40, and D‐17 cell lines were concomitantly treated with verdinexor and doxorubicin at fixed ratios of their IC_50_ values for 96 hours. Cell proliferation was measured using the CyQUANT assay and the CI was calculated using the Chou‐Talalay method.^32^ Canine OS cells treated in combination with doxorubicin and verdinexor showed reduced proliferation when compared with cells treated with verdinexor or doxorubicin alone (Figure 5A). Additionally, calculated CIs were synergistic (CI < 1) at all combinations tested, with the exception of one combination in the D‐17 cell line (Figure 5B). These data support the notion that combinational treatment of verdinexor and doxorubicin in canine OS cell lines works synergistically to reduce cell proliferation.

### 3.6 Verdinexor impairs DNA damage repair caused by doxorubicin treatment

**FIGURE 6:**
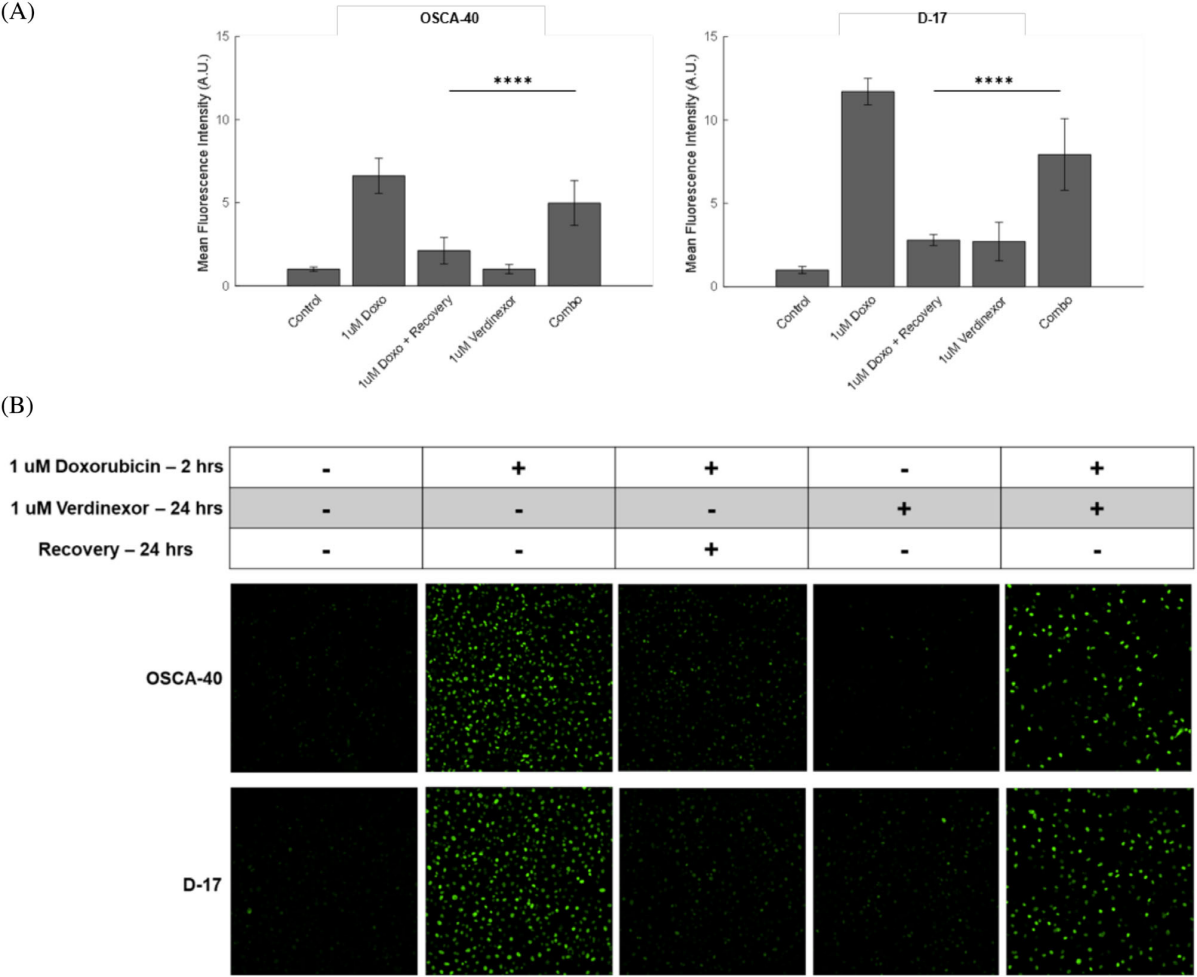
Verdinexor inhibits repair of DNA damage induced by doxorubicin in canine osteosarcoma (OS) cell lines. γH2A.X expression, a marker for DNA damage, was detected via immunofluorescence in OSCA‐40 and D‐17 cells. (A) Cells were either untreated (control) or treated with 1 μM doxorubicin for 2 hours. Cells treated with doxorubicin were washed and allowed to recover in normal media (1 μM Doxo + Recovery) or 1 μM verdinexor (Combo) for 24 hours. Data were analysed using a two‐tailed Student's *t*‐test. ****P < .0001 and presented as mean ± SD. (B) Representative semi‐quantitative images of γH2A.X expression. DNA damage because of doxorubicin persisted longer when recovered in verdinexor. Verdinexor alone did not induce DNA damage [Colour figure can be viewed at wileyonlinelibrary.com]

To further elucidate a mechanism by which verdinexor and doxorubicin work synergistically to reduce OS cell proliferation, the canine D‐17 and OSCA‐40 cell lines were treated with media either containing 0.1% DMSO, 1 μM doxorubicin for 2 hours, 1 μM verdinexor for 48 hours, or 1 μM doxorubicin for 2 hours followed by recovery in 1 μM verdinexor or 0.1% DMSO for 24 hours. Cells were then stained for γH2A.X, a marker for DNA damage. Previous studies demonstrate that SINE compounds in combination with doxorubicin or other topoisomerase II inhibitors work synergistically by inducing more DNA damage via nuclear retention of topoisomerase II or by inhibiting repair of damaged DNA caused by DNA damaging agents through downregulation of DNA repair proteins.^37^, ^38^, ^39^ We assessed the impact of verdinexor on cellular DNA damage repair and found in that cells treated with doxorubicin for 2 hours and recovered in verdinexor, γH2A.X expression was increased compared with cells recovered in media without verdinexor (Figure 6). These data suggest that verdinexor impairs DNA damage repair processes as treatment with verdinexor alone did not induce DNA damage. This may provide a partial explanation for the synergism observed in canine OS cell lines treated with the combination of verdinexor and doxorubicin (Figure 5).

## 4 DISCUSSION

There is significant need for effective therapeutic strategies in the treatment of canine OS as >90% of dogs succumb to metastatic disease that is refractory to chemotherapy. Recent attempts to improve outcomes in OS patients with single‐agent targeted molecular therapeutics and immunotherapy has proved largely unrewarding.^6^, ^13^ As systemic chemotherapy remains the standard treatment for metastatic disease, novel therapeutic strategies such as the addition of small molecule inhibitors to cytotoxic chemotherapy may enhance their overall efficacy, ultimately improving survival outcomes. One family of novel compounds, termed SINE, inhibit the major nuclear export protein Exportin 1 (XPO1), which is frequently overexpressed in cancer.^14^, ^16^, ^17^, ^18^, ^19^, ^20^, ^21^, ^22^ SINE compounds work synergistically with standard cytotoxic agents to enhance cancer cell death.^37^, ^39^ Verdinexor (KPT‐335) is a SINE compound developed for companion animal use that is safe and tolerable in canines at a biologically active dose of 1.5 mg/kg three times a week.^23^ Preliminary evidence of the in vitro activity of verdinexor has been documented in several canine cancer cell lines and verdinexor exhibits biologic activity in canine lymphoma patients; however, XPO1 expression and therapeutic targeting of XPO1 in canine OS has not been evaluated.^23^, ^28^, ^29^ We therefore sought to characterize the expression of XPO1 in canine OS tumours and OS cell lines to determine if XPO1 inhibition represents a viable target for therapeutic intervention and determine the activity of verdinexor alone or in combination with doxorubicin in canine OS cell lines as a prelude to future clinical trials in dogs.

XPO1 transcript and protein were overexpressed in the majority of canine OS tumours and cell lines as compared with normal canine osteoblast cells. Concordant with our data, XPO1 is overexpressed in human OS compared with normal tissues.^19^ Similarly, XPO1 is overexpressed in a number of human cancers such as gastric, oesophageal, bladder, and multiple myeloma and increased XPO1 expression has been associated with poor survival outcome.^22^, ^34^, ^35^, ^36^ Although the prognostic impact of XPO1 expression in canine OS was not evaluated in this study, we demonstrated that XPO1 overexpression is common event in canine OS cells and as such, XPO1 may represent a relevant target for therapeutic intervention. Treatment with verdinexor significantly decreased cell proliferation in canine OS cell lines at low nanomolar IC_50_ concentrations (range 30 ‐ 74 nM); however, sensitivity to verdinexor did not directly correlate with basal XPO1 expression levels. Prior studies evaluating the XPO1 inhibition in a panel of sensitive and resistant cell lines found that intrinsic cellular resistance was not attributed to XPO1 occupancy by selinexor, suggesting that cancer cell responsiveness to XPO1 inhibition depends on the modulation of signalling pathways downstream of XPO1.^40^ In contrast, canine osteoblast cells were markedly less sensitive to verdinexor as demonstrated by an IC_50_ value of 21 μM. Importantly, the canine OS cell line IC_50_ concentrations were physiologically relevant as previous verdinexor pharmacokinetic profiles established a C_max_ of 253 ng/mL (or 572 nM) in dogs receiving oral verdinexor.^29^

Verdinexor decreased OS cell proliferation which correlated with modest, but statistically significant dose‐dependent increases in caspase‐3/7 enzymatic activity in canine OS cell lines. No differences in caspase‐3/7 enzymatic activity were detected in normal osteoblasts at both concentrations of verdinexor tested. Although these data demonstrate that canine OS cells were significantly more sensitive to the effects of verdinexor in an apoptotic manner, the minimal to mild increases in caspase‐3/7 activity in OS cells treated with verdinexor (1‐10 μM) were discordant given the low nanomolar IC_50_ concentrations of verdinexor. These findings suggest that decreased OS cell proliferation with verdinexor treatment is primarily attributed to cytostatic and/or cytotoxic effects rather than apoptosis. Additional experiments are necessary to determine the mechanism by which verdinexor inhibits proliferation in OS cell lines.

To determine the effects of verdinexor on XPO1 inhibition in canine OS cells, we evaluated XPO1 transcript and protein expression 24 hours following treatment with verdinexor. We observed decreased XPO1 expression in all four cell lines at 0.1 and 1 μM verdinexor when compared with vehicle treated cells with the exception of the Abrams cell line. At 0.1 μM, Abrams cells expressed more XPO1 than vehicle treated cells; however, at 1.0 μM, XPO1 expression was reduced. Prior in vitro studies have documented that cellular exposure to SINE compounds leads to a time‐dependent accumulation of nuclear proteins; however, the nuclear protein abundance progressively decreases as a consequence of enhanced ubiquitination and proteasome‐dependent XPO1 degradation.^15^ As such, it is possible that in Abrams cells treated with verdinexor at 0.1 μM, XPO1 function is inhibited as evidenced by a compensatory increased in XPO1 mRNA, but not ubiquinated for proteasome degradation. XPO1 transcript was elevated in all cell lines at both concentrations of verdinexor consistent with a compensatory response to XPO1 inhibition, a finding similarly seen in canine melanoma cells.^28^

To determine if a combinational approach would enhance the chemotherapeutic efficacy of doxorubicin, a topoisomerase II inhibitor routinely used in the treatment of canine and human OS, OS cell lines were treated with verdinexor and doxorubicin and potential synergism was assessed. Studies performed in human multiple myeloma and acute myeloid leukaemia cell lines demonstrate therapeutic synergistic effects using a combination of selinexor and topoisomerase II inhibitors.^37^, ^38^, ^39^ Concordantly, we found that combination treatment with verdinexor and doxorubicin in three canine OS cell lines worked synergistically, causing a significant decrease in cell proliferation compared with either drug alone. Reported mechanisms for this synergism include enhanced DNA damage through nuclear retention of topisomerase IIα (TopoIIα) and reduction of DNA damage repair proteins as a result of XPO1 inhibition.^37^, ^38^, ^39^, ^41^ Doxorubicin is a TopoIIα poison which prevents topoisomerase re‐ligation of cleaved DNA resulting in double‐stranded DNA breaks.^42^ Via the forced nuclear retention of TopoIIα by XPO1 inhibition, the efficacy of doxorubicin is enhanced as a result of increased DNA damage leading to cellular apoptosis.^38^, ^41^ Alternatively, pre‐treatment with DNA damaging agents like doxorubicin, followed by recovery in selinexor prolonged the DNA damage repair process resulting in increased cytotoxicity and apoptosis.^39^

To evaluate the mechanism by which doxorubicin and verdinexor work synergistically in canine OS cells, cells were pre‐treated with doxorubicin to induce DNA damage and recovered in media with or without verdinexor for 24 hours. To visualize DNA damage, we used the γH2A.X marker, a histone that is phosphorylated in response to double‐stranded DNA breaks.^43^ In health, γH2A.X forms within minutes of an insult to DNA and can recede to basal levels by 24 hours. Persistent γH2A.X may represent unrepairable double‐stranded breaks.^43^ When OS cells were treated with doxorubicin and recovered in normal media for 24 hours, γH2A.X returned to near basal levels. In contrast, when OS cells were recovered in verdinexor for 24 hours following treatment with doxorubicin, γH2A.X foci persisted, suggesting a delay or failure to repair damaged DNA. Importantly, treatment with verdinexor alone did not induce γH2A.X foci. These findings are consistent with data generated in the U2OS human OS cell line.^39^ Collectively, these findings suggest that verdinexor alters DNA damage repair processes in vitro and this may, in part, represent a mechanism by which doxorubicin and verdinexor work synergistically in canine OS cells.

## 5 CONCLUSION

In summary, XPO1 is a relevant target for therapeutic intervention in canine OS as XPO1 is overexpressed in canine OS tumours and cell lines. Inhibition of XPO1 using the novel orally bioavailable XPO1 inhibitor, verdinexor results in inhibition of cellular proliferation in canine OS cell lines at biologically achievable concentrations. Verdinexor upregulated XPO1 mRNA while downregulating XPO1 protein, indicative of on‐target drug effects. Combinatorial treatment using doxorubicin and verdinexor demonstrated synergistic activity in canine OS cells, possibly through hindrance of the DNA damage repair process. Together, these findings provide important pre‐clinical data supporting future clinical investigations of verdinexor in combination with doxorubicin dogs with OS.

## CONFLICT OF INTEREST

The authors declare no potential conflict of interest.

## Data Availability

The datasets used and/or analysed during the current study are available from the corresponding author on reasonable request.
